# Supplementary material for: Prevalence of stunting and its associated factors among children 6–59 months of age in pastoralist community, Northeast Ethiopia: A community-based cross-sectional study
Source: PLoS One. 2022 Feb 3;17(2):e0256722. doi: 10.1371/journal.pone.0256722 (PMC8812981; doi:10.1371/journal.pone.0256722)
Supplement: S1 Table — (DOCX) [file pone.0256722.s001.docx]

**Qafar Afah tan esseroora /Afar version questionnaires/**

Xubti Daqaara

- 1. Awda migaaqa :**_________**

0.2 Makafta migaaqa:__________

0.3 Esserok baxsa le bed loowo /______/______/

**Table 8. hayto Raawa: Xaagoyse Gufnea /Interviewer Visit/**

|  | Gufne /Visit/ 1 | Gufne /Visit/ 2 | Gufne /Visit/ 3 |
| --- | --- | --- | --- |
| Ayro |  |  |  |
| Xaagoyse |  |  |  |
| Xalu |  |  |  |

0.4 Xalu /Result**/**

1. Gabkaleh

2. Raddi geyam maxiqiyyo

3. Cineenih

4. Qaxak gaba kaleh

5. Kalah

0.5 Xagoyse**:**

Bedu__________

Migaaqa ____________________________

0.6 Xagoyso edde takke ayro:_______________

0.7 Diggosê migaaqa: _____________________

Feerâ warah asta ________ Ayro__________

|  |  |  |  |
| --- | --- | --- | --- |

Mabla garay edde qibbim wakti /udduuru

Qimbisna

Miraceta

Foyyah yan araca kib

**1 ^hayto^  Exxa: Sahdisso kee ayyuntiino qidaddoh rakisso**

Table 9. 10hayto Raawa: Sahdisso kee ayyuntiino qidaddoh rakisso urri xaltanik Qafara Rakaakayak Awsi Rasu /1/ Xubti Daqaara Akda baxiso/ Caxa alsa 2010

| Loowo | Essero | Bedu daffeesaanam | Amo kor /Skip/ |
| --- | --- | --- | --- |
| 100 | Buxa abba miyyaay? | 1. Awkî abba inna 2. Awkî ina 3. Kalah |  |
| 101 | Awkî inak karma magidey?  (sanatal) | __________ |  |
| 102 | Awkî ina Rahim caagid manna tanih? | 1. Digibteh tan  2. Madagibin  3. Baxsimeenih yanin  4. Cabento  5. Abuura  6. Kalah (Baxsale caalata) ___________________ |  |
| 103 | Ani agatat raqatah? | 1.Qafara  2. Amhara  2. Oromo  3. Tigray  4. Kalah (baxsale caalata)  99. Gacsa mali |  |
| 104 | Diini kok macay? | 1. Ortoxokis  2. Muslim  3. Protestant  4. katoliik  5. kalah |  |
| 105 | Buxah addal karmak 5 qunxa urri magide yakkeh? | _______________ |  |
| 106 | Inak aw awka qarise xagarak barittok magide leh? | 1. Yaktubeh yakriyem maxiqa  2.Yaktubeh yakriyem xiqah (madab sin baritto)  3. 1^hato^ baritto  4. 2 ^hayto^ caddo baritto  5. Fayyo le caddoh baritto |  |
| 107 | Awkî xaltani aw qarisek mataama le? | 1. Buxah inna  2. Buqreh xiina  3. Dacrsittoh xiine  4. Tellemo abe  5. Kalah^a^ |  |
| 108 | Buxah abba taamah maca lee? | 1. Buxah ina(buxa barra)  2. Buqreh xiina  3. Dacrsittoh xiina  4. Tellemo abe  5. Kalah^a^ |  |
| 109 | Itta buxah qadad magidey? | ___________ |  |
| 110 | Buxah addal karma akak 10 sanatak guba akak takke, taamit xiqe waamara kee karamak 60 daga akak takke mari buxah addal qadaadak magidev yakkeenih? | ___________ |  |
| 111 | Buxah addal maali cato abtam miyaay? | 1. Mangom barra  2. Mangom baqla  3. Dubuh baqla  4. Nammay inkih |  |
| 112 | Saqa litoo? | 1. Yeey 2. Baleey |  |
| 113 | Buxa caddol gaalak magiide litoh? | __________ |  |
| 114 | Buxah abba^h^ Buqrek iro alsal ittat geyan culenti magidey? (Itiyopia Lakqoh) | ___________ |  |
| 115 | Buxah abba^c^ alsal ittat buqrek magide yakke culenta litoh? | ­________________ |  |
| 116 | Buxah addal ubconto magide litoonuh? | _________ |  |
| 117 | Buqre ardi lito? | 1. Yeey 2. Baleey |  |
| 118 | Buxah caddol magide yakke hektaara litoonuh ? | ______ |  |
| 119 | Buxah caddol magide yakke hektaara litoonuh ? | _____ |  |
| 120 | Softineetit may cattimtaa? | 1. Yeey 2. Balee |  |

Others^a^ = Numti amo cuggaysot tamlisen mara, doolat temlo le maray, barteeniti, NGO taama abeenit.

Households^b^= Saqah xiina, Saqi murtixiina, buqreh caxaaxuwa kee qelboh xiina

Households^c^= Fokkaaqo elle aban araca, mango maray buqrek iro taamita axcih sokkar cuggaysot dacayri yan maray, kasel aba maray, ramad cata maray saqi tellemo aba mara.

**2 ^hayto^ Exxa: Urri caalata kee urru elle qarisan inna**

**Table 10. hayto Raawa: Karamak 6-59 alsat yan urri caalat kee urru elle qarisan inna Qafar Rakaakayak Awsi Rasuk Xubti Daqaari addal akda Baxiso /Caxah alsa 2010**

Ta esseroora gacisa xaltani akke waytek awka haanam angu elle yaaxennak xabba haanamay illa taham fan kaa yaaxigem faxximta.

| **Loowo** | **Essero** | **Bedi Weelo** | **Amo kor /Skip/** |
| --- | --- | --- | --- |
| 200 | Awkî qunxa saqal aw maqanxa yellek karma kak magidey? (alsa) | 1. Labih 2. 2. Sayyo |  |
| 201 | Awkî yoobokeh yanwaqdi inki saaqatih addat maca kaa teceenih? | _________ |  |
| 202 | Awkî magideh caddoy akah yoobokem? | 1. 1^st^ 2. 2^nd^ 2. 3^rd^ 4. > 4^th^ |  |
| 203 | Karmah kok qunxa saqal tellek yoobokem? | 1.<24 2. 24-48  3. >48 4.Karmah kak qunxah alqi maayu |  |
| 204 | Awkî mannal yoobokeh? | 1. Tiyah  2. Gangaytu |  |
| 205 | Awki ankle yoobokeh? | 1. budah addal ullat ina gabal yooboke 2. Budah addal qafioyat mihratlih Baleey  3. Qafiyat hakral yooboke |  |

**3 ^hayto^ Exxa: Urri caalata kee urru elle qarisan inna**

Table 11. hayto Raawa: Karamak 6-59 alsat yan urri caalat kee urru elle qarisan inna Qafar Rakaakayak Awsi Rasuk Xubti Daqaari addal akda Baxiso /Caxah alsa 2010

Ta esseroora gacisa xaltani akke waytek awka haanam angu elle yaaxennak xabba haanamay illa taham fan kaa yaaxigem faxximta.

| **Loowo** | **Essero** | **Bedi Weelo** | **Amo kor /Skip/** |
| --- | --- | --- | --- |
| 300 | kulli waqdi angu aaxuk suge? | 1. Yeey 2. Baleey |  |
| 301 | Awkî yooboke waqdi makina saaqatak wadri angu yeexeh? | 1. Tunnaluk 2. 1-2 Saaqata wadir 3. Ayro sugeek gera |  |
| 302 | Awkî yabukay sidiica ayroh addat anguk iro tan maaqo kah mayteceenih? | 1. Yeey 2. Baleey |  |
| 303 | Awkî yoobokeh yanwaqdi inki saaqatih addat maca kaa teceenih? | 1. TÛ macinno 2. Lee 3. Mutuku 4. Malaba 5. Laa/gaali/wadar/ can 6. Kalah tanim |  |
| 304 | Awkî inah angu aaxuk magideh alsasugeh? | 1. < 4  2. 4-6  3. >6   1. 88. Maaxiga |  |
| 305 | Awkî aham fana angu yaaxe? | 1. Yeey 2. Baleey |  |
| 306 | 208 essero balee intek, awkî angu akah aaxewaam maca? | 1. Inah qafiyat taqabiy  2. Awkî isih yirgiqeh  3. Idqino sabbata |  |
| 307 | 208 essrol balee intek,Awkî angu yaaxem soolise wak mannal sooliseh? | 1. Qaxaqaxa 2. Inkinnah sooliseh |  |
| 308 | Awkî inah angu soolise waqdi alsakmagidet sugeh? | _____________ |  |
| 309 | Awkî namma ayrok (48 saaqatak afal) afal ossotina maaqo yokmeh maysugeh? | 1. Yeey 2. Baleey | Taesserok “Baleey “, intek ta esserok 310-312 amo kor |
| 310 | Awkî inah angu kala ossatina maaqo yakmem magideh alsat qimbise? | __________ |  |
| 311 | Warrayteh tan 24 saaqatak afal,awkî ossotinah riyo le aw gibdi maaqok makina uddur yokmeh ? | 1. < 3 udduuru  2. 3 udduuru  3. > 3 udduuru |  |
| 312 | Taturteh tan 24 saaqatih addat awkîh maaqo afeenalmay xageh? | 1. Yeey 2. Baleey |  |
| 313 | Awkî maaqo yakme waqdi macal akmuk sugeh? | 1. Cayyari  2. Kalaasa  3. Malkaqa  4. Gabah |  |
| 314 | Taturteh tan 24saaqatih addat awkî caxâ miiru may yokmeeh? | 1. Yeey 2. Baleey |  |
| 315 | Taturteh tan 24 saaqatih addat awkî caxaaxuwa may yokmeh sugeh? | 1. Yeey 2. Baleey |  |
| 316 | Taturteh tan 24 saaqatih addat can nakeh may suge? | 1. Yeey 2. Baleey |  |
| 317 | Taturteh tan 24 saaqatih addat laana yokmeh may sugeh? | 1. Yeey 2. Baleey |  |
| 318 | Taturteh tan 24 saaqatih addat, cado yokmeh may sugeh? | 1. Yeey 2. Baleey |  |
| 319 | Taturteh tan 24 saaqatih addat, qelbo kee wonnah tan maaqo xageh may sugeh? | 1. Yeey 2. Baleeyah |  |
| 319.1 | Sidukeesiratan tan 24 saaqatih addat, qelbo kee wonnah tan maaqo xageh may sugeh? | 1. Yeey 2. Baleeyah |  |
| 320 | Tatureteh tan 24 saaqatih addat ayooxin qasbo xageeh may sugeh? | 1. Yeey 2. Baleeyah |  |
| 321 | Awkî aftabi caagi manna le? (yellek yoo uysubulley)? | 1. Aftabu mali 2. Gudaagude le (yakeesen aftabu yoh abeh)   Aftabuk gabkaleh |  |
| 322 | Awkî xaltani aw taynabo, urri qarisiyyi aydakaakan /fayo/ may beyteh sugteh? | Yes 2. Baleeyah |  |
| 323 | Awkî lakbat aftabi mudum aracah (BCG) mayleh? | 1. Yeey 2. Baleeyah |  |
| 324 | Taturteh tan 6 alsih addat, bagi alluwa kalalu takkeh tan diwa beh may suge? | 1. Yeey 2. Baleeyah   1. 88. Maxiga |  |
| 325 | Awkî vaytaami A beh may sugeh? (dayli efeena ken ublus)? | 1. Yeey 2. Baleeyah   1. 88. Maaxiga |  |
| 326 | Taturteh tan namma ayyaamat, awkî lakmiseh maysugeh? | 1. Yeey 2. Baleeyah |  |
| 327 | Awkî lakmisah yan waqdi qaafiyat harka kaa maaybetaanah? | 1. Yeey 2. Baleeyah |  |
| 328 | Sinil xayih tan qaafiyat harka sin buxak magide xeerih? (gexa gaco) | _____/______ |  |
| 329 | Awkî lakmiseh may sugeh? | 1. Qaso 2. Bagi biyaaaka 3. anxeexu 4. Bagi gexo   Gosonu |  |
| 330 | Warrayteh tan nammay ayyaamah ubkâ warakata xexxar (baarah) kee luftok(cayla) magide yakkeh? | ________ |  |
| 331 | Awkî/â xexxar(\|) kee baar(̶) qedaalis (cm) |  |  |
| 331 | Awkî/â lufto (cayla) qedaalis /kg/ | ________ |  |
| 333 | Record MUAC ( Mid Upper Circumference) | _________ |  |
| 334 | Does the child have sign of odema/ Bilateral oedema present (circle +/ or ++ or ++, if yes) | 1. Yeey (+/++/+++)  2. Baleeyah |  |
| 335 | Is there sign of kwashiorker | 1. Yeey 2. Baleeyah |  |

**4^hayto^ Exxa: xaltanih caalat qaafiyat ayfaafih doqaso**

Table 12. hayto Raawa: Qafar Rakaakayak Xubti Daaqara xaltani caalt kee qaafiyat ayfaafa dooqayso akda Baxiso/ Caxah alsa 2010

| **Loowo** | **Essero** | **Caddo bedu** | **Amokor** |
| --- | --- | --- | --- |
| 400 | Iná cisaare baxa xalte wak karamak magidet sugteh? | _________ |  |
| 401 | Awkî ina sittat makina baxa leh ? | _______ |  |
| 402 | Awkî ina soonibit wak aw xalte waqdi ossotinah afto maygeyteh? | 1. Yeey 2. Baleeyah |  |
| 403 | Awkî mankel yoobokeh? | 1. 9 gubay (<9) 2. 9 alsa 3. 9 alsak daga |  |
| 404 | Awkî ina xaltaamak nahart aw xaltek wadir qaafiyat harkal maakina wak is fokkaqteh? | 1. 1 2. 2  3. 3 4. 4 |  |
| 405 | Xalay soloh daylat tintifiqeh maytaaxigeh? | 1. Yeey 2. Baleeyah |  |
| 406 | Manna yan xalay sooloh diwaatat doqaysimteh taaxigeh? | 1. Xalay hooroh kaniima /Pills / 2. Irba (Depo-Provera) 3. kalah^d^ |  |
| 407 | awkî ina soonibah maytan? | 1. Yeey 2. Baleeyah |  |

kalah^d^ =maganat han mansaf (norplant), konxom (condom) kee ayro loowak.

**5^hayto^ Exxa: Saytunaany, buxa marih saytunaane caalata**

Table 13. hayto Raawa: Qafar rakaakay awsi rasu xubti daqaarak buxa marak Akda baxiso /Caxah alsa 2010

| **No.** | **Questions** | **Coding Classification** | **Skip** |
| --- | --- | --- | --- |
| 500 | Edde tantifiqeh tan leek raceena ankey? | 1. Waqaytu 2. Buyyi 3. Qeela 4. Pumpo |  |
| 501 | Lee bahtoonuh maakina wakti sinik beytah (gexak gacol) | ______ |  |
| 502 | Buxa mari saytu leet dooqaysimaana^c?^ | 1. Yeey 2. Baleeyah |  |
| 503 | 502hayto essero yeey gacissek leet elle dooqaysimaanam mannay | 1. Laqisak 2. Keemikalat saytunnoosak 3. Sarol ayxuxxuuruk 4. Salalisak |  |
| 504 | Buxa mari ayroh addat leek makina liitirit yantifiqen | ____________ |  |
| 505 | Warrayteh tan 3 alsat sugteh tan bagi gexoh(atet)buxal baaceh mari mayyan? | 1. Yeey 2. Baleeyah |  |
| 506 | Aalsih addat ku buxah marak bagi gexo baaceh suge mari may yan | ___________ |  |
| 507 | Inkih yan buxa mari bicsen daacoh buxat yantifiqe? | 1. Yeey 2. Baleeyah |  |
| 508 | Gaba kalqissan waqdi mannal doqaysimtaanah (tantifiqeenih)? | 1. Leet tiyah 2. Saabun kee kalah tan saytunossa iko 3. Saabut tiya |  |
| 509 | Buxa mari mannah tan quduufa kalaanamih maknayat doqoysimaanah edde | 1. Buuxah yan araca caxan 2. Boodoh adda 3. Quduufa booduy kulli num edde caxa 4. Darqih edde yantifiqen (composting) 5. Cararrisiyya |  |
| 510 | Sin awda aw makafta daacok saytuni? | Yeey 2. Baleeyah  88. maaxiga |  |
| 511 | Sin caafatal qaso maytan? | 1. Yeey 2. Baleeyah |  |
| 512 | 408 esserok yeey intek awkî ina soonibah sugte waqdi qaso teetit macayteh? | 1. Yeey 2. Baleeyah |  |

Sin awda aw makafta daacok saytuni= dariifa deesaasak dariifa saytunaane kee ayyunti daacoh buxa kee numti amo daacoh buxa tanim kee kulli buxa gaba elle kalqisah tan aracah loonum keenik cubbus.
