# Supplementary material for: Prevalence of stunting and its associated factors among children 6–59 months of age in pastoralist community, Northeast Ethiopia: A community-based cross-sectional study
Source: PLoS One. 2022 Feb 3;17(2):e0256722. doi: 10.1371/journal.pone.0256722 (PMC8812981; doi:10.1371/journal.pone.0256722)
Supplement: S2 Table — (DOCX) [file pone.0256722.s002.docx]

**English Version Questionnaires**

- 1. **Name of Kebelle:_________**
  2. Name of village:__________

0.3 Questionnaire identification number /_____/_______/

**Table 7. Interviewer Visit**

|  | Visit 1 | Visit 2 | Visit 3 |
| --- | --- | --- | --- |
| Date |  |  |  |
| Interviewer |  |  |  |
| Result |  |  |  |

0.4 **Result**

1. Completed

2. Respondents not available

3. Refused

4. Partially completed

5. Other

05 **Interviewer:**

Code: __________

Name: ____________________________

06 Date of interview:_______________

07 Checked by (Supervisor/Investigator) Name: _____________________

Signature________ Date __________

|  |  |  |  |
| --- | --- | --- | --- |

Time at the beginning of interview

Let’s begin.

**Instructions**

Fill in the blank space

**Section 1 Demographic and Socio-Economic Characteristic**

Table 8. Demographic and socio-economic characteristics of children families at Dubti district, Zone 1, Afar region, January/February 2018

| **No.** | **Questions** | **Coding Classification** | **Skip** |
| --- | --- | --- | --- |
| 100 | Who is head of the household? | 1. Father of the child 2. Mother of the child 3. Other*, specify___________ |  |
| 101 | What is the age of the mother of the child (in years)? | __________ |  |
| 102 | What is the mother’s current marital status? | 1. Married  2. Single  3. Separated  4. Divorced  5. Widowed |  |
| 103 | To which ethnic group do you belong? | 1. Afar 2. 2. Amhara   3. Oromo  4. Others** |  |
| 104 | What is your religion? | 1. Orthodox  2. Muslim  3. Protestant  4. Catholic  5. Other (specify)_____ |  |
| 105 | How many under 5 years’ children are there in the HH? | _________________ |  |
| 106 | What is the current educational level of the mother/care taker for the child? | 1. Unable to read and write  2.Unable to read and write (Informal education)  3. Primary education  4. Secondary education  5. Higher education |  |
| 107 | What is the current occupation of mother/ care taker? | 1. Housewife only  2. Agro-pastoralist  3. Pastoralist  4. Merchant/ Trade  5. Others^a^ |  |
| 108 | What is the current occupation of husband? | 1. Housewife only  2. Agro-pastoralist  3. Pastoralist  4. Merchant/ Trade  5. Others^a^ |  |
| 109 | What is your current total family size? | ___________ |  |
| 110 | What number of children below 10 years, disabled members and elders above 60 years are there in the HH? | 1. Children below 10 years___ 2. People with special needs____ 3. Elders above 60 years 4. Total _________ |  |
| 111 | Who is decision making on use of money in the household? | 1. Mainly spouse  2. Mainly Husband  3. Only husband  4. Both jointly |  |
| 112 | Do you have livestock? | 1. Yes 2. No |  |
| 113 | What is the number of camel per household? | ____________ |  |
| 114 | What is the average **monthly** **on-farm** income of the households^b^? (in ETB) | _______________ |  |
| 115 | What is the number of shoat per household you have? |  |  |
| 116 | What is the number of cow/ ox per household you have? |  |  |
| 117 | Do you have currently irrigated land? | 1. Yes 2. No |  |
| 118 | How much hectare of irrigated land per the household do you have? | _________1 hectare |  |
| 119 | What is the average **monthly** **off-farm** income of the households^c^? (in ETB) | ______________  Mention the majour income sources:______________________ |  |
| 120 | Are you PSNP user? | 1. Yes 2. No |  |

*Care givers of the targeted child, ** Tigre, Wolayita

Others^a^ =, Somali Private organization employee, government employee, student, NGO employee

Households^b^= Livestock, livestock products, vegetables and maize are the major source of on-farm income in the study area.

Households^c^=In the study area, the major non-farm activities were employment as guard and laborer in the *Tendaho* Sugar Factory irrigation canal construction, charcoal making, gift from their relatives, and livestock trading.

**Section 2 Child Characteristics**

Table 4. Characteristics of children aged 6-59 months at Dubti district, Zone 1, Afar region, January/ February 2018

If the respondent is not the mother, ensure that the respondent knows the breastfeeding practices between birth and now.

| **No.** | **Questions** | **Coding Classification** | **Skip** |
| --- | --- | --- | --- |
| 200 | What is the sex of the child? | 1. Male 2. Female |  |
| 201 | What is the age of the child? (in **Months)** |  |  |
| 202 | What is the birth order of the targeted child? | 1. 1^st^ 2. 2^nd^   3. 3^rd^ 4. > 4^th^ |  |
| 203 | If the targeted child does have younger sibling, what is the age of the younger sibling in months? | 1. <24 2. 24-48  3. >48 4. It has no younger siblings |  |
| 204 | What was the type of birth you/ the mother had with this child? | 1. Single  2. Twin |  |
| 205 | Where was the child born?/ Place of delivery/ | 1. At home assisted with traditional birth 2. At home assisted with health professional 3. Health facility/ Skilled birth attendant |  |

**Section 3 Child Carrying Practice**

Table 5. Characteristics of children aged 6-59 months at Dubti district, Zone 1, Afar region, January/ February 2018

If the respondent is not the mother, ensure that the respondent knows the breastfeeding practices between birth and now.

| **No.** | **Questions** | **Coding Classification** | **Skip** |
| --- | --- | --- | --- |
| 300 | Was this child ever breastfed? | 1. Yes 2. No |  |
| 301 | How many hours after birth was the child first given the breast/ Initiation of breastfeeding of child? | 1. Immediately 2. After 1 to 24 hrs 3. After a day |  |
| 302 | Was the child given any substance other than breast milk during the first 3 days of life/ Child received pre-lactation food or fluid? | 1. Yes 2. No |  |
| 303 | What type of pre-lactation food/fluids kind the child was provided with? | 1. None 2. Water 3. Butter 4. Honey/ Water with sugar 5. Cow/camel/goat milk 6. Other (Specify) |  |
| 304 | For how many MONTHS this child has been getting Exclusive Breastfeeding (EBF)? | 1. < 4  2. 4-6  3. >6  88. I don’t know |  |
| 305 | Is the child still breast feeding? | 1. Yes 2. No |  |
| 306 | If response to question number 305 is “No”, what is the reason for not breast feeding? | 1. Maternal health problem  2. Refusal of child  3. Maternal pregnancy |  |
| 307 | If response question number 305 is “No”, what is the weaning status of the child? | 1. Partial 2. Full |  |
| 308 | What was the age of the child when its breast feeding stopped (in months)? | __________ |  |
| 309 | Did the child receive complementary food for last 48hrs in addition to Breastfeeding? | 1. Yes 2. No | If “No”, skip to question number, 310-312. |
| 310 | What was the child’s age when it started complementary feeding (in months)? | _______ |  |
| 311 | How many times was the child fed mashed or pureed, or solid or semi-solid food during the last 24 hours/ Frequency of feeding/day? | 1. < 3 times  2. 3 times  3. > 3 times |  |
| 312 | Is the child bottle fed during the last 24 hours? | 1. Yes 2. No |  |
| 313 | What is/ was the method of feeding the child has been used? | 1. Bottle  2. Cup  3. Spoon  4. Hand |  |
| 314 | Did the Child intake fruit during the last 24 hours | 1. Yes 2. No |  |
| 315 | Did the Child intake vegetable during the last 24 hours? | 1. Yes 2. No |  |
| 316 | Did the Child intake milk during the last 24 hours? | 1. Yes 2. No |  |
| 317 | Did the Child intake egg during the last 24 hours? | 1. Yes 2. No |  |
| 318 | Did the Child intake meat during the last 24 hours? | 1. Yes 2. No |  |
| 319 | Did the Child intake beans & other legumes? | 1. Yes 2. No |  |
| 320 | Did you use iodized salt during the last 24 hours? | 1. Yes 2. No |  |
| 321 | What is the child’s immunization status? (*Ask to see the vaccination card*))? | 1. Not vaccinated 2. Partially vaccinated 3. Fully vaccinated |  |
| 322 | Has the caretaker or the mother ever taken any component of health service training/counseling on a child care practice issue? | 1. Yes 2. No |  |
| 323 | Observe BCG scar on the upper arm. Does the scare exist? | 1. Yes 2. No |  |
| 324 | Has the child received deworming medicine in last 6 months? | 1. Yes 2. No  88. I don’t know |  |
| 325 | Has the child received vitamin A supplementation in the last 6 months? (show capsule) | 1. Yes 2. No  88. I don’t know |  |
| 326 | Has the child been sick in the last two weeks? | 1. Yes 2. No |  |
| 327 | Does the child visit health institutes while it has ever sick or caught by diseases? | 1. Yes 2. No |  |
| 328 | How far is the nearby health institution from your home in km and hour? (round trip) | _____/________ |  |
| 329 | Has the child have illness? /Ask all questions for one illness, then for the next illness/ | 1. Malaria 2. Stomach 3. Measles 4. Diarrhea 5. Cold/ Pneumonia/ Whooping cough |  |
| 330 | How many times in the last 6 months has the child’s weight and or length/height been recorded on the growth card? (see the card) | ________ |  |
| 331 | Record length/height of the child (in cm) | _________ |  |
| 332 | Record weight of the child (in kg) | _________ |  |

**Section 4 Maternal Characteristics and Health Service Utilization**

Table 6. Maternal characteristics of Dubti District, Zone 1, Afar region, January/ February 2018

Others^d^ =Norplant, condom and calendar methods

| **No.** | **Questions** | **Coding Classification** | **Skip** |
| --- | --- | --- | --- |
| 400 | What was the age of the mother at first birth (years)? | ____________ |  |
| 401 | What is/are total number of children born to a mother? | ___________ |  |
| 402 | Was the mother given with extra meal during pregnancy or lactation? | 1. Yes 2. No |  |
| 403 | What was the gestational age at birth? | 1. Less than 9 months  2. At 9 months  3. Greater than 9 months |  |
| 404 | How many times the mother of the child visited health facility for ante natal care follow-up (ANC)/ post-natal care follow-up (PNC)? | 1. 1 2. 2 3. 3 4. 4 |  |
| 405 | Do you/ the mother of the child know family planning methods? | 1. Yes 2. No |  |
| 406 | What type of family planning have you/ she ever used? | 1. Pills 2. Depo-Provera 3. Others^d^ |  |
| 407 | Is the mother of the child currently pregnant? | 1. Yes 2. No |  |

**Section 5 Environmental Health Characteristics of Households**

Table 7. Environmental health characteristics of household of Dubti district, Zone 1, Afar region, January/February 2018

| **No.** | **Questions** | **Coding Classification** | **Skip** |
| --- | --- | --- | --- |
| 500 | What is the main source of drinking water you are using? | 1. River 2. Unprotected spring water 3. Protected spring water 4. Public tap |  |
| 501 | What time you spent to obtain/fetch drinking water? (round trip) | __________minutes |  |
| 502 | Do the HH use treated water by any means^e^ | 1. Yes 2. No |  |
| 503 | If the response to question number 502 is yes, what method are using to get treated water? | 1. Boiling 2. Chlorine 3. Cloth 4. Filtration 5. We do not use anything else |  |
| 504 | What amount of water is the HH used per day (liters)? | _____________ |  |
| 505 | Did you face episodes of acute water diarrheal (AWD) to any of your family member during the past 3 months? | 1. Yes 2. No |  |
| 506 | When did you/ your family members face episodes of acute water diarrheal (AWD)? ( in Months) | _____________ |  |
| 507 | Do you have latrine available and used for all HH members? | 1. Yes 2. No |  |
| 508 | What materials are you using to wash hands after toile? | 1. Using water only 2. Using soap sometimes 3. Using soap always |  |
| 509 | What method of waste disposal is the HH using? | 1. Open field disposal 2. In a pit 3. Common pit 4. Composing 5. Burning |  |
| 510 | Is your Kebelle/ village free of ODF^f^? | 1. Yes 2. No   88. I don’t know |  |
| 511 | Is there malaria in this village? | 1. Yes 2. No |  |
| 512 | If response for question number 511the is “Yes”, did mother have malaria during her pregnancy of this child? | 1. Yes 2. No |  |
