# Supplementary material for: Prevalence of stunting and its associated factors among children 6–59 months of age in pastoralist community, Northeast Ethiopia: A community-based cross-sectional study
Source: PLoS One. 2022 Feb 3;17(2):e0256722. doi: 10.1371/journal.pone.0256722 (PMC8812981; doi:10.1371/journal.pone.0256722)
Supplement: Attachment — Submitted filename: comments.docx [file pone.0256722.s004.docx]

**Prevalence of stunting and its associated factors among children 6-59 months of age in pastoralist community, Northeast Ethiopia: A community based cross sectional study**

**Reviewer comments:**

1. The authors need to drastically revamp the grammatical aspect of the article, especially in the ‘Introduction and background’.
2. The authors need to specify what new findings are coming up in the study which are affecting stunting among the subjects, as compared to extant literature. So, the ‘discussion’ needs to focus more on what has already been found and how the current study adds on to the existing body of work, as opposed to just finding similarities with existing hypotheses.
3. Concrete measures as to the alleviation of the existing situation and policy perspectives need to be focused on. The last two lines of the article seemed to touch upon the topic, but no sound policy recommendations were made on the basis of the study.
4. How the current situation affects the SDG attainment of the country as a whole, the relevant topic was mentioned in the introduction, but it was not followed up in the conclusion and discussion.
5. The analysis, specification of the data, methods and the tables are fine, but the background and the interpretation of the study need to be solidified.
