# Supplementary material for: Prevalence of stunting and its associated factors among children 6–59 months of age in pastoralist community, Northeast Ethiopia: A community-based cross-sectional study
Source: PLoS One. 2022 Feb 3;17(2):e0256722. doi: 10.1371/journal.pone.0256722 (PMC8812981; doi:10.1371/journal.pone.0256722)
Supplement: Attachment — Submitted filename: Authors response_stunting.docx [file pone.0256722.s005.docx]

**Author’s response for reviewers**

| **No** | **Referee** | **Comment** | **Response** | **Reference** |
| --- | --- | --- | --- | --- |
| 1 | Editor | Please ensure that your manuscript meets PLOS ONE's style requirements, including those for file naming. The PLOS ONE style templates can be found at  <https://journals.plos.org/plosone/s/file?id=wjVg/PLOSOne_formatting_sample_main_body.pdf> and  <https://journals.plos.org/plosone/s/file?id=ba62/PLOSOne_formatting_sample_title_authors_affiliations.pdf> | We have adhered to the style of PLOS ONE’s accordingly. | It can be appreciated form the entire manuscript. |
| 2 | Editor | We note that [Figure(s) 1] in your submission contain [map/satellite] images which may be copyrighted. All PLOS content is published under the Creative Commons Attribution License (CC BY 4.0), which means that the manuscript, images, and Supporting Information files will be freely available online, and any third party is permitted to access, download, copy, distribute, and use these materials in any way, even commercially, with proper attribution. For these reasons, we cannot publish previously copyrighted maps or satellite images created using proprietary data, such as Google software (Google Maps, Street View, and Earth). For more information, see our copyright guidelines: <http://journals.plos.org/plosone/s/licenses-and-copyright> | Since we are unable to get permission from the copyright holder, we have removed the figure from the manuscript. We believed that description we provide in the manuscript is sufficient to define the study area. | Page 6, Line 121-124. |
| 3 | Editor | In your Data Availability statement, you have not specified where the minimal data set underlying the results described in your manuscript can be found. | We have uploaded the data set, according to the journal requirement. |  |
| 4 | Editor | Please provide a copy of your questionnaire in the original language and English and, as Supporting Information. | We have provided accordingly the questionnaire. |  |
| 5 | Editor | We suggest you thoroughly copyedit your manuscript for language usage, spelling, and grammar. If you do not know anyone who can help you do this, you may wish to consider employing a professional scientific editing service. | We have edited the language accordingly. | It can be seem from the manuscript. |
| 6 | Editor | We noticed you have some minor occurrence of overlapping text with the following previous publications, which needs to be addressed:  - <https://bmcnutr.biomedcentral.com/articles/10.1186/s40795-019-0300-0> - <https://journals.plos.org/plosone/article?id=10.1371%2Fjournal.pone.0195361> - <https://mrmjournal.biomedcentral.com/track/pdf/10.1186/s40248-019-0188-1.pdf>  In your revision ensure you cite all your sources (including your own works), and quote or rephrase any duplicated text outside the methods section. Further consideration is dependent on these concerns being addressed. | We have edited the manuscript to avoid the text overlapping with previous published works. We have made the necessary corrections by appropriate citation, quoting, and rephrasing the text. | It can be seen from the manuscript. |
| 7 | Reviewer #1 | The authors need to drastically revamp the grammatical aspect of the article, especially in the ‘Introduction and background’. | The language was extensively edited. | You can see the whole manuscript. |
|  | Reviewer #1 | The authors need to specify what new findings are coming up in the study which are affecting stunting among the subjects, as compared to extant literature. So, the ‘discussion’ needs to focus more on what has already been found and how the current study adds on to the existing body of work, as opposed to just finding similarities with existing hypotheses. | Thank you for the comment. We have tried to focus on the major findings and its implication with local context. | The discussion section. |
|  | Reviewer #1 | Concrete measures as to the alleviation of the existing situation and policy perspectives need to be focused on. The last two lines of the article seemed to touch upon the topic, but no sound policy recommendations were made on the basis of the study. | Thank you for the comment. Based on the findings, we have provided plausible recommendations. Especially targeting underlying factors for stunting, following organized and collaborative approach in addressing stunting, and use of the local context, since the community is nomadic. | Page 24: Line 401-417. |
|  | Reviewer #1 | How the current situation affects the SDG attainment of the country as a whole, the relevant topic was mentioned in the introduction, but it was not followed up in the conclusion and discussion. | Thank you for the comment, we have addressed the issue in the discussion and conclusion part of the manuscript accordingly. | Discussion and conclusion part |
|  | Reviewer #1 | The analysis, specification of the data, methods and the tables are fine, but the background and the interpretation of the study need to be solidified. | Thank you for the comment. We have tried to strengthen the background and the result part, based on the comments given. |  |
| 9 | Reviewer #2 | In the results section – while presenting the results, present only proportions, not the numbers of participants, avoid presenting both numbers as well as percentages. Readers can check it out the numbers if they want from the Tables. If it is presented in numbers, then, it is highly challenging for readers to understand the results. | Thank you for the comment, we have corrected accordingly. | Result section |
| 10 | Reviewer #2 | In Table 2 information of morbidity among children has presented but not been discussed, the result shows one-fifth of children has two or more diseases, and it is important to discuss here, 20% is quite a large number. | Thank you for the comment, we have addressed discussed the morbidity result and its implication for nutritional intervention also indicated. | Page 20, Line 326-320. |
| 11 | Reviewer #2 | Since the information of Diseases among children has been collected and has used in Table 2 as well, it is also advisable to use it in logistic regression and see whether the diseases will have any role to play in stunting among children. | We have checked the association of morbidity and stunting. However, it was not significant in the X^2^ test and we didn’t include in the logistic regression. |  |
| 12 | Reviewer #3 | 1. The article needs copy editing with regard to language and ambiguity of points. For example, the sentence “The odds of stunting was increased, so does age of the child increased as compared to 6-11 months of children.” And at line 39. “………..livelihood strategies remain largely dependent on the climate system and susceptible to seasonal” at line 89 has an abrupt ending. | Thank you for the comment, we have addressed the issue accordingly by undergoing through review of the manuscript. | It can be seen from the entire manuscript.  The specific comments were also addressed, example Page 1, Line 70. |
| 13 | Reviewer #3 | 2. It was mentioned that total 555 households were interviewed (there were total 909 children from 554 households as can be seen from the analysis in table 1). How did you choose the children from these household what was the exclusion inclusion criteria? What was the method adopted to select the child? This need to be mentioned in the methodology part. | Thank you for the comment, If a household had two or more eligible children, the index child or the youngest one was included in the study. We have included this information in the manuscript accordingly. | Page 7, Line 148-150. |
| 14 | Reviewer #3 | 3. Definition of fully, partly and not vaccinated should be mentioned in footnotes. As per WHO “children aged 12–23 months, if the final primary vaccination is at 9 months of age – this is the most commonly chosen target population”. Therefore, it would be better to exclude children who have not completed 12 months from the analysis of immunization. | Thank you for the comment. We have provided the definition in the method section. Since, we define the status of vaccination by completing all doses of vaccine for the given age as fully vaccinated. Missing at least one dose of vaccine for the given age as partially vaccinated and not taking any doses of vaccine for the given age as not vaccinated. We believe excluding the children who have not completed 12 months is not important. | Page 8, Line 182-187. |
| 15 | Reviewer #3 | 4. Under diseases in table 2 what are the childhood diseases covered in the study. A description of the diseases included in the study would be useful for the prospective reader. | We have included the description of the diseases found in the study, accordingly. | Page 12, Line 238-241. |
| 16 | Reviewer #3 | 5. Table no 3 there is row on decision making – “Decision making on use of money”. The answers are ‘mainly spouse” and “mainly husband”. If you interviewed the mother for information what would option “mainly spouse” mean, it would mean husband. These options need to be looked at. | Thank you for the concern, spouse in this study was to mean wife. To avoid the confusion we have use the word wife instead. | Table 3. |
| 17 | Reviewer #3 | 6. In the discussion part at line 321 there is a sentence “Policies seeking to increase the number of animals in the nomadic culture are therefore crucial in combating malnutrition. Increasing animal health by raising awareness about the prevention and control of animal diseases, improving access to animal health facilities and, most importantly, piling up animal fodder is necessary.” Could not understand how that’s relevant here. | Thank you for the comment. The reason why we included the statement was due to the fact that economic status measured by Tropical Live Stock (TLU) was found to be one of the correlates of stunting. Households who were poor in economic status were at higher risk of stunting. Since the study was conducted among nomadic community who are dependent on animas as livelihood, we have stressed the significance of awareness creation to improve animal health and providing sufficient fodder to increase the size of animal population. |  |
| 18 | Reviewer #3 | 7. The referencing style is not uniform in the paper. | We have used the reference style recommended by the journal and we have amended the irregularity in the style. |  |
