# Supplementary material for: Prevalence of stunting and its associated factors among children 6–59 months of age in pastoralist community, Northeast Ethiopia: A community-based cross-sectional study
Source: PLoS One. 2022 Feb 3;17(2):e0256722. doi: 10.1371/journal.pone.0256722 (PMC8812981; doi:10.1371/journal.pone.0256722)
Supplement: Attachment — Submitted filename: Review report1.docx [file pone.0256722.s006.docx]

Thank you for sharing the revised version. The author has addressed the comments satisfactorily. However, one more concern indicated below need to be addressed.

On page 8, In the definition of fully vaccinated- children who had received a vaccination against tuberculosis (BCG), three doses each of the DPT and polio vaccines, and a measles vaccination by 12 months of age…… In standard practice, it considers the children aged 12 to 23 months for full immunisation; even WHO also suggests the period 12-23 months to calculate full immunisation….it is better to follow the standard practice so the results can also be comparable with other studies as well.
