# Supplementary material for: Prevalence of stunting and its associated factors among children 6–59 months of age in pastoralist community, Northeast Ethiopia: A community-based cross-sectional study
Source: PLoS One. 2022 Feb 3;17(2):e0256722. doi: 10.1371/journal.pone.0256722 (PMC8812981; doi:10.1371/journal.pone.0256722)
Supplement: Attachment — Submitted filename: Response to Reviewers.docx [file pone.0256722.s007.docx]

**Manuscript title:** Prevalence of stunting and its associated factors among children 6-59 months of age in pastoralist community, Northeast Ethiopia: A community-based cross-sectional study

**Response to reviewers**

| **No** | **Referee** | **Comment** | **Response** | **Reference** |
| --- | --- | --- | --- | --- |
| 1 | Editor | Please review your reference list to ensure that it is complete and correct. If you have cited papers that have been retracted, please include the rationale for doing so in the manuscript text, or remove these references and replace them with relevant current references. Any changes to the reference list should be mentioned in the rebuttal letter that accompanies your revised manuscript. If you need to cite a retracted article, indicate the article’s retracted status in the References list and also include a citation and full reference for the retraction notice. | We have reviewed the reference list and it is correct. Retracted papers not cited in this manuscript.  We have changed reference number 22 in page 8. The former reference in the old version of the manuscript was unpublished work. We have replaced it with published articles.  The reference number 30 and 31 in old version were the same reference, but appears as different references in the manuscript. This issue was addressed accordingly. |  |
| 2 | Reviewer #3 | The manuscript requires copy editing of the language and better presentation. The authors may seek professional help if needed. | Thank you very much for the comment. Copy editing of the language has been extensively done accordingly. | It can be seen from the entire manuscript. |
| 3 | Reviewer #3 | If Line 95 "The global nations' sustainable development goals (SDGs) " it should be "the United Nation's SDGs....." | Thank you. We have corrected it accordingly. | Page 1; Line 95. |
| 4 | Reviewer #3 | Table no 1 :"Educational status of a mother" it should be "Educational status of mother" | We have corrected it accordingly. | Table 1. |
| 5 | Reviewer #3 | Line 244 &b 245 "80.5% of the majority of children were breastfed exclusively for at least six months" It should be "majority (80.5%) of the children were breastfed exclusively for at least six months". In many places use of parenthesis is missing in the manuscript. | Thank you very much for the comment. It has been modified accordingly. Throughout the manuscript such kind of corrections have been made. | Page 13; Line 247. |
| 6 | Reviewer #3 | Please cross check the figures in the table and the in the description. In a few places the figures in description are mismatching with what is in the table. For example; in line 240 "This study showed that 56.5% of the children were " but the table 1 says the percentage is 56.3 and in Line no 260 "households concerning the use of money (71.2%)" Table 3 shows this to be 71.3 %. Such mistakes should be avoided. | Thank you very much. We have made the corrections accordingly. Now the figures in the text description and in the respective tables are speaking the same. |  |
| 7 | Reviewer #3 | Under morbidity, what are the diseases covered in the study are still not mentioned anywhere in the paper. | Thank you for the comment. We have addressed the issue accordingly. | Page 13; Line 253-255. |
| 8 | Reviewer #3 | On page 8, In the definition of fully vaccinated- children who had received a vaccination against tuberculosis (BCG), three doses each of the DPT and polio vaccines, and a measles vaccination by 12 months of age…… In standard practice, it considers the children aged 12 to 23 months for full immunisation; even WHO also suggests the period 12-23 months to calculate full immunisation….it is better to follow the standard practice so the results can also be comparable with other studies as well. | Thank you very much for the comment. We have modified the definition accordingly. The status of vaccination has been computed according to the WHO standard, by considering the age group from 12-23 months. | Page 8; Line 182-189. Table 2. |
